# A PHLID Model for Tomato Bacterial Canker Predicting on Epidemics of the Pathogen

**DOI:** 10.3390/plants12112099

**Published:** 2023-05-25

**Authors:** Akira Kawaguchi, Shoya Kitabayashi, Koji Inoue, Koji Tanina

**Affiliations:** 1 Western Region Agricultural Research Center (WARC) (Kinki, Chugoku, and Shikoku Regions), National Agriculture and Food Research Organization (NARO), Fukuyama 721-8514, Hiroshima, Japan; 2 Research Institute for Agriculture, Okayama Prefectural Technology Center for Agriculture, Forestry and Fisheries, Akaiwa 709-0801, Okayama, Japan; 3 Okayama Agriculture Development Institute, Akaiwa 701-2221, Okayama, Japan

**Keywords:** *Clavibacter michiganensis* subsp. *michiganensis*, incubation period, epidemiology, simulation model, primary and secondary transmissions

## Abstract

A pathogen, healthy, latently infected, infectious, and diseased plant (PHLID) model for botanical epidemics was defined for tomato bacterial canker (TBC) caused by the pathogenic plant bacteria, *Clavibacter michiganensis* subsp. *michiganensis* (*Cmm*). First, the incubation period had to be defined to develop this type of model. To estimate the parameter of incubation period, inoculation experiments were conducted in which it was assumed that infection is transferred to healthy plants by cutting with contaminated scissors after cutting infected plants with early symptoms or symptomless. The concentration of *Cmm* was increased over 1 × 10^6^ cells/g plant tissue at 20 cm away from the inoculated point on the stem 10 days after inoculation, and then the approximate incubation period of TBC in symptomless infected plants was defined as 10 days. The developed PHLID model showed the dynamics of diseased plants incidence and fitted the curve of the proportion of diseased plants observed in fields well. This model also contains the factors of pathogen and disease control, and it was able to simulate the control effects and combined two different control methods, which were the soil and scissors disinfections to prevent primary and secondary transmissions, respectively. Thus, this PHLID model for TBC can be used to simulate not only the increasing number of diseased plants but also suppressing disease increase.

## 1. Introduction

Tomato bacterial canker (TBC) is a major threat to tomato production worldwide, caused by *Clavibacter michiganensis* subsp. *michiganensis* (*Cmm*) [1,2,3,4,5,6]. *Cmm* causes substantial economic loss worldwide [1,2,3,4,5,6]. Sources of primary *Cmm* inoculum include naturally infected seeds, infected transplants, or infected plant debris in the soil [2,3,7,8,9]. Secondary spread of the pathogen is caused by water splashes, non-sterilized pruning scissors, or workers’ hands [2,3,10,11,12]. *Cmm* is exuded through hydathodes in guttation fluid under high humidity conditions, such as those found inside greenhouses [12].

In commercial greenhouses, the number of diseased plants increases rapidly after pruning [2,11]. Therefore, the authors have previously verified that infected plant debris acts as the primary inoculum, and unwittingly pruning latently infected tomato plants without disinfection serves as a source of secondary spread, which occurs sooner than the spread from infected debris in commercial greenhouses in Japan [2,9]. To prevent TBC outbreaks, it is crucial to disinfect agricultural equipment, such as pruning shears and gloves, before pruning [2,9]. However, despite such efforts, TBC continues to occur globally [1,3,13]. Therefore, a comprehensive understanding of TBC epidemics is necessary to effectively prevent future outbreaks.

Previously, the authors reported that the healthy–latently–diseased (HLD) model, which was developed for TBC epidemics and can simulate the increase in the number of diseased plants and the duration of disease incidence, was based on the theory of the susceptible–infected–recovered (SIR) model [3,14]. In the field of botanical epidemiology, SIR and the susceptible–exposed–infected–recovered (SEIR) models have been commonly used to understand the mechanisms of plant disease epidemics [3,15,16,17].

In general, the SIR model is used to model diseases in which individuals have permanent immunity or at least a very long period of temporary immunity, while the SEIR model is used to model diseases with a long incubation period and a long period of immunity [18,19,20]. The authors developed the HLD model for TBC epidemics, but this model did not take the incubation period into account [3]. Furthermore, the factor of primary pathogen should be considered in the epidemic model because the infection mechanism of plant diseases such as TBC involves both primary and secondary inoculum and spread [16]. To gain a more precise understanding of the epidemics of TBC in commercial greenhouses, a new epidemic model should be developed.

Thus, the objectives of this study were to develop a new epidemic model called the pathogen–healthy–latently–infectious–diseased (PHLID) model, based on the SEIR theory of TBC. We aimed to analyze the relationship between disease incidence and the probability of infection from primary sources such as seeds or soil, as well as secondary sources such as pruning with infected scissors. Additionally, we added a new parameter to estimate the control effects of preventing primary and secondary infections. To develop the PHLID model, we assessed the incubation periods of *Cmm* in latently infected tomato plants.

## 2. Materials and Methods

### 2.1. Incubation Period of Cmm in Latently Infected Tomato Plants

The *Cmm* strain CMM16-3 [3], which is pathogenic to tomato (*Solanum lycopersicum* cv. Momotaro), was used in this study. Cell suspensions of the *Cmm* strain were prepared from cultures grown on potato dextrose agar medium for 48 h and adjusted to an OD_600_ of 0.01 (1 × 10^7^ cells/mL). Scissors were dipped in the cell suspension for 1 s, and 50 tomato plants (2 months old) growing in plastic pots with soil (20 cm depth × 9 cm diameter; 1 plant per pot) were inoculated by pruning two compound leaves. Those plants were grown in a greenhouse at 25–30 °C. To confirm infection, stem samples were collected from five asymptomatic plants at 0, 1, 3, 7, and 10 days after inoculation (dai). The stem samples were collected from the inoculated point of the plant (0 cm), as well as three parts of the stem located 10, 20, and 40 cm away from the inoculated point (0.2 g fresh weight per plant, one sample per plant).

The population of *Cmm* in inoculated plants was assessed using the serial dilution plate method [3,14]. First, the samples were initially washed with sterile distilled water and subsequently crushed in 1 mL of sterile distilled water using an autoclaved mortar and pestle. Then, 10-fold serial dilutions (100 μL) of the samples were plated and spread onto *Cmm*-selective medium SMCMM [2,3,21], following which the plates were incubated at 25 °C for 5 days. The colony growth was monitored on five plates for each dilution, and the numbers of colony-forming units (CFUs) were determined. To verify the identity of the suspected colonies grown on SMCMM plates, ImmunoStrip for Cmm (Agdia, Elkhart, IN, USA), a rapid immune-chromatographic strip test, was used [22]. The bacterial populations in plants (CFU/g of plant tissue) were log_10_-transformed before statistical analysis. This assay was independently performed twice. Statistical analysis was performed using the RStudio user interface (RStudio, Inc., version 1.2.5001) for R software (R Foundation for Statistical Computing, version 3.6.1). ANOVA and Tukey’s HSD tests (*n* = 10, 5 plants per experiment) were conducted using RStudio.

### 2.2. PHLID Model, Basic Reproduction Number (R_0_), and Effective Reproduction Number (R_t_)

**Figure 1:**
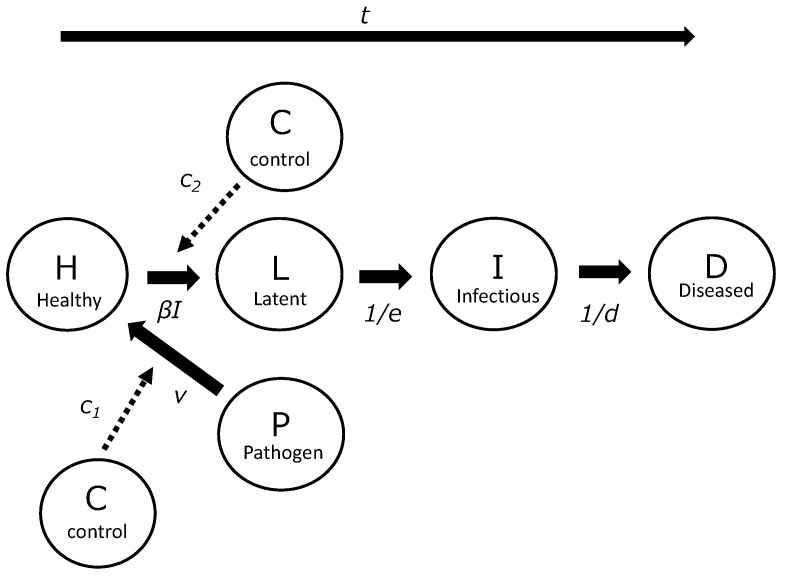
This schematic diagram represents the PHLID (pathogen, healthy, latently infected, infectious, and diseased) disease model, which illustrates the dynamics of tomato plants from the healthy (*H*) to latently infected (*L*), infectious (*I*), and ultimately to diseased (*D*) stages with symptoms of TBC. It also depicts the production of inoculum or pathogen individuals (*P*) and any control effects (*C*), particularly the interruption of primary and secondary inoculum transmissions (*c*_1_ and *c*_2_).

In this study, the PHLID model was defined based on the SEIR model. The TBC system was represented by a set of linked differential equations for a compartmental system, describing the changes in the status of plants from healthy (*H*) to latently infected (*L*), infectious (*I*), and ultimately to diseased (*D*) with symptoms of TBC, such as canker, wilt, and dead plants (Figure 1). Growers in commercial greenhouses often cut infectious latently infected tomato plants without visible symptoms [8]. In this study, latently infected plants (*L*) were defined as non-infectious and symptomless plants and assumed to be infected by soil or seed-borne pathogens. Infectious plants (*I*) were defined as plants that were infectious and still symptomless, and ultimately diseased plants (*D*) were completely wilted and dead plants and often removed by farmers to avoid touching [2,3,9,11]. Generally, although plants showing symptoms of disease can be infectious, farmers tend to refrain from touching visibly diseased plants and instead remove them from the greenhouse [2,3,9,11]. In the common commercial greenhouses in Japan, wind was blocked, and the soil was covered with plastic films over tubes to avoid water splashing during irrigation. Although insects might be possible vectors of *Cmm* infection, there is currently no evidence to support this. The fertilization method used was irrigation using water mixed with nutrients. Therefore, these factors were not considered in this study [2,11]. Although plant debris from *Cmm*-infected and/or dead plants inside the soil can become the primary inoculum for soil infection in the next cultivation year [2,9], this study only considers one term of cultivation, from planting seedlings to the end of cultivation. Therefore, the interactions between the causal infections described in this study were absent. Thus, in this study, *D* was defined as post-infectious individuals.

In this study, post-infectious individuals were defined as *R*. Comparing the definitions of PHLID and SEIR, *H*, *L*, *I*, and *D* correspond to *S*, *E*, *I*, and *R*, respectively. However, the PHLID model differs from the original SEIR model. Madden and van den Bosch [16] showed that the botanical SEIR model was created by adding new parameters of primary inoculum (*P*) and primary infection rate (*v*) (Figure 1). *P* represents the amount of inoculum such as spores, bacterial cells or other infectious units, or amount of inoculum, although the precise amount of inoculum is often unknown in commercial fields [16]. For the purposes of this study, *P* was set at 1.0. Furthermore, in this study, new original parameters were defined to simulate any control effects, specifically the interruption of primary and secondary inoculum transmissions (*c*_1_ and *c*_2_) (Figure 1). Thus, the general forms of the equations are given by:

*dH/dt* = −*vSPc*_1_ − *βSIc*_2_, (1)

*dL/dt* = *vSPc*_1_ + *βSIc*_2_ − *L(*1*/e)*, (2)

*dI/dt* = *E(*1/*e)* − *I(*1*/d)*, (3)

*dR/dt* = *I(*1/*d)*, (4)

*β* = *Bk*, (5)

*N* = *H* + *L* + *I* + *R* = 1 (6)

*D* = *I* + *R* (7)

in which *N* is the proportion of tomato plants in total (=1, or 100%), *D* is diseased plants, *β* is the infection rate of plants, *B* is the infection rate per contact, *k* is the number of contacts per day, *d* is the duration of infection (days), 1/*d* is the removal rate of susceptible plants, *e* is the rate of latent individuals becoming infectious (i.e., the incubation period), and 1/*e* is the average incubation period.

Some of the parameters used in this study were taken from previous reports (Table 1) [3,9,23,24], while others, such as 1/*e*, were determined by the experiment conducted in this study (Table 1). The parameter *v* represents the primary infection rate and includes *v_seed_* and *v_soil_*, which are the infection rates from infected seeds and soil infected by infected plant debris, respectively. Additionally, the model includes two parameters, *c*_1_ and *c*_2_, which represent the control effects against primary and secondary inoculum, respectively. These parameters were defined using the integrated relative risk (IRR) obtained through meta-analysis procedures (Table 1) [3,24]. If the IRR value is 0.1, the control treatment can decrease to 10% of that of the non-treatment, and the control effect is considered extremely high in the greenhouse. If no diseased management is conducted, *c_1_* and *c_2_* are given a neutral value of 1.0. However, if diseased management is conducted, *c_1_* and *c_2_* are given values of 0.48 and 0.12, respectively (Table 1). The *c*_1_ value was obtained from the IRR value of the control effect by soil disinfection treatment using commercial dazomet (C_5_H_10_N_2_S_2_) micro granule [24], while the *c_2_* value was obtained from the IRR value of the disinfection for contaminated scissors by dipping them into a 70% ethanol solution [3].

To compare the PHLID model with the actual dynamics of TBC incidences previously reported, disease incidence data from six different commercial greenhouses (with over 1000 tomato plants per greenhouse), which experienced an outbreak of TBC in Okayama, Japan in 2005–2006 [2,3,11], were utilized. The actual dynamics of TBC incidences in seven different commercial greenhouses in Okayama from 2005 to 2008 were also used, where soil disinfection treatment was conducted using commercial dazomet (C_5_H_10_N_2_S_2_) micro granules, and scissors and groves were disinfected using 70% ethanol isolation [2,11,24].

Moreover, the basic reproduction number (*R*_0_) and the effective reproduction number (*R_t_*) of TBC were calculated. The equations for *R*_0_ and *R* were presented below:

*R*_0_ = *(vc*_1_ + *βc*_2_*)/(*1/*d)*, (8)

*R_t_* = *SR*_0_, (9)

In epidemiology, *R*_0_ refers to the expected number of cases directly generated by one infected individual in a population where all individuals are susceptible to infection. On the other hand, *R_t_* refers to the number of cases generated in the current state of a population [25]. In commonly used infection models, the infection can spread in a population when *R*_0_ or *R_t_* > 1, but not when *R*_0_ or *R_t_* < 1. *R*_0_ is constant, while *R_t_* is variable and can gradually or rapidly decrease as the disease progresses. Generally, a higher *R*_0_ value makes it harder to control the epidemic. Based on the parameters of the PHLID model, both *R*_0_ and *R_t_* were estimated.

## 3. Results

### 3.1. Population Dynamics of Cmm in Latently Infected Tomato Plants and Incubation Period

**Figure 2:**
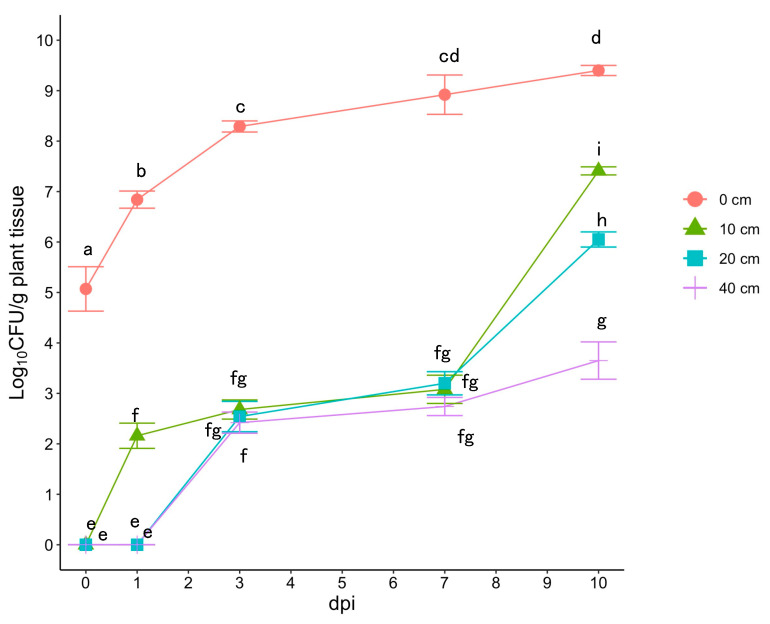
Migration and population dynamics of *Cmm* at different sites on tomato plants. The cells of *Cmm* were detected from the inoculated point of the plant (0 cm) and three parts of the stem located 10, 20, and 40 cm away from the inoculated point. The error bars represent the 95% confidence interval (CI) range. Different letters indicate a significant difference between the bars with different concentrations of *Cmm* (*p* ≤ 0.05, Tukey’s HSD test).

At the inoculation point (0 cm) of latently infected tomato plants, a *Cmm* population of 5.07 (95% CI: 4.63–5.51) log_10_ CFU/g plant tissue, which is approximately 1.0 × 10^5^ CFU/g plant tissue, was detected immediately after inoculation (0 dpi) (Figure 2). The *Cmm* population continued to increase, and at 10 dpi, 9.40 (95% CI:9.30–9.50) log_10_ CFU/g plant tissue was detected at the inoculation point (0 cm) (Figure 2). In contrast, no *Cmm* cells were detected at three points on the stem located at 10, 20, and 40 cm away from the inoculation point at 0 dpi. However, *Cmm* populations of approximately 3.0 log_10_ CFU/g plant tissue were detected at these points at 7 dpi (Figure 2). At a point on the stem located 10 cm away from the inoculation point at 10 dpi, a *Cmm* population of 7.41 (95% CI: 7.33–7.49) log_10_ CFU/g plant tissue, which is approximately 2.6 × 10^7^ CFU/g plant tissue, was detected (Figure 2). At a point on the stem located 20 cm away from the inoculation point at 10 dpi, a *Cmm* population of 6.05 (95% CI: 5.90–6.20) log_10_ CFU/g plant tissue, which is approximately 1.0 × 10^6^ CFU/g plant tissue, was detected (Figure 2). According to Kawaguchi et al. [3], a *Cmm* population of over 1.0 × 10^6^ CFU/g plant tissue can cause TBC symptoms. Therefore, the incubation period of TBC was assumed to be 10 days.

### 3.2. Development of PHLID Model

**Figure 3:**
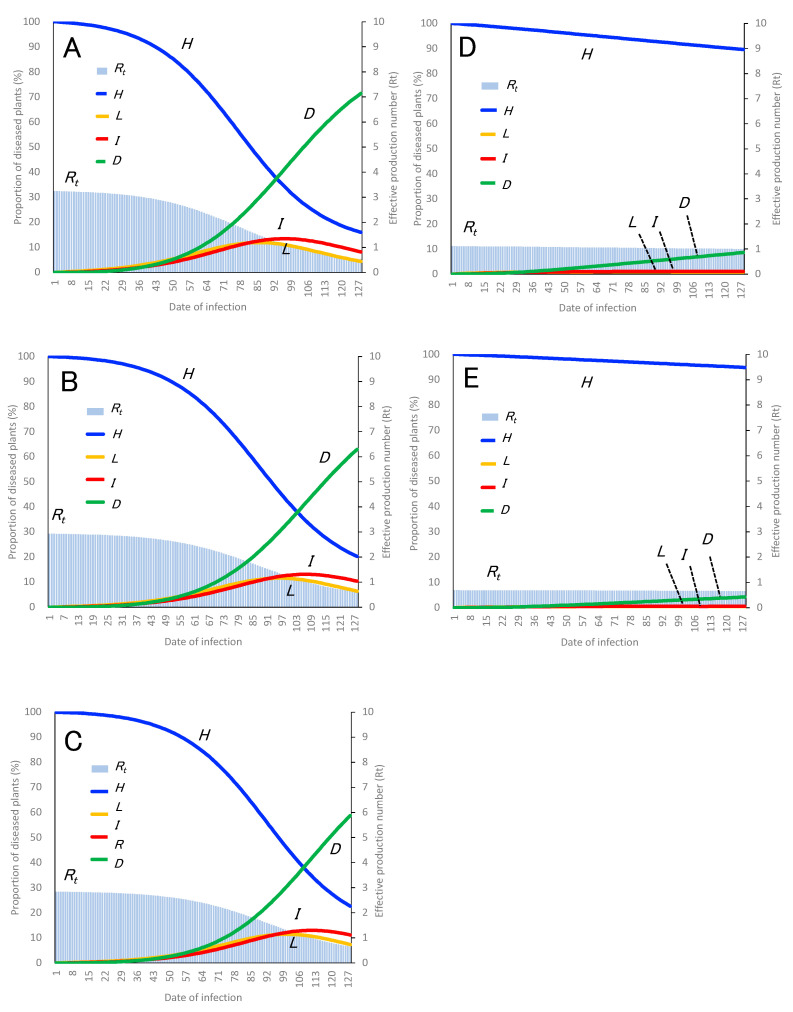
Development of PHLID model for TBC transmission estimation is depicted in this figure. (**A**) Model A illustrates the dynamics of the effective reproduction number (*R_t_*) of TBC for soil infection. (**B**) Model B shows the dynamics of Rt for seed infection. (**C**) Model C estimates the values of *H*, *L*, *I*, *D*, and *R_t_* of TBC in the case of soil disinfection based on model A. (**D**) Model D estimates the same values in the case of scissors disinfection based on model A. (**E**) Model D also estimates the values in the case of combined contaminated soil and scissors disinfection based on model A.

The parameters used to develop the PHLID model of TBC were estimated and defined using data obtained from this study and several reports (Figure 2 and Table 1). Based on the variables listed in Table 1 (*B* = 0.706, *d* = 12, *k* = 0.286, 1/*e* = 10, *v_soil_* = 0.067, and *c*_1_ = *c*_2_ = 1 for the no disease control situation), the dynamics of healthy plants (*H*), latently infected plants (*L*), infectious plants (*I*), diseased plants (*D*), and *R_t_* are presented in PHLID model A for soil infection (Figure 3A). In the case of seed infection, a different type of PHLID model (model B) is presented based on the variables listed in Table 1 (*B* = 0.706, *d* = 12, *k* = 0.286, 1/*e* = 10, *v_seed_* = 0.04, and *c*_1_ = *c*_2_ =1) (Figure 3B).

**Figure 4:**
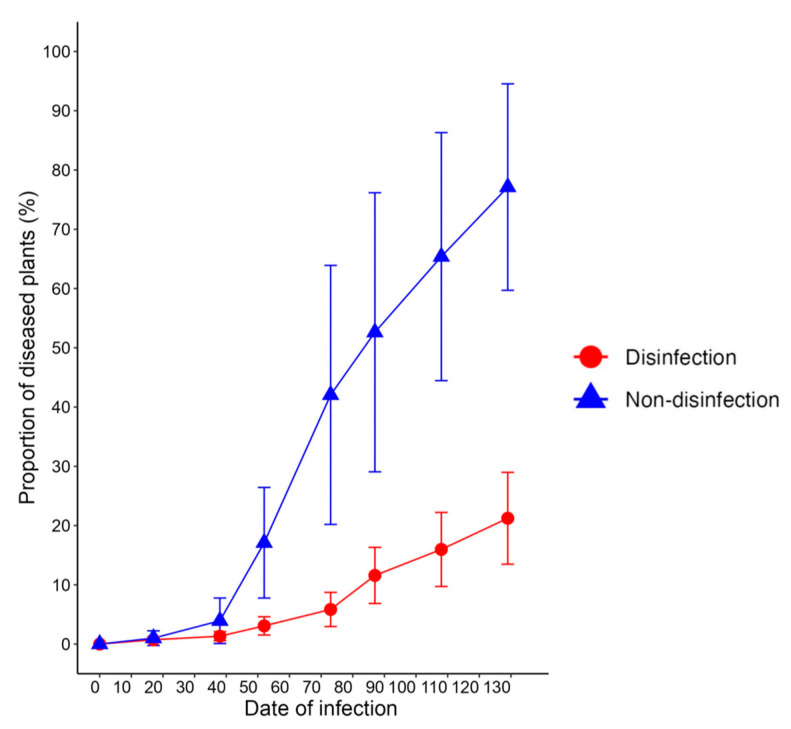
TBC incidence dynamics observed in commercial greenhouses are presented. Two types of greenhouses are compared: non-disinfection and disinfection. The latter refers to the average population disease incidence (%) in commercial greenhouses where soil disinfection treatment was conducted using commercial dazomet micro granules, and scissors and gloves were disinfected using 70% ethanol isolation.

In model A, *R*_0_ was estimated as 3.2, and it could take up to 103 days from the beginning of transmission to decrease by *R_t_* < 1.0 (as shown in Figure 3A). In model B, *R*_0_ was estimated as 2.9, and it could take up to 110 days from the beginning of transmission to decrease by *R_t_* < 1.0 (Figure 3B). Although the disease incidence level of model A is slightly higher than that of model B, both models are similar. When comparing the two types of PHLID models (A and B) with the observed disease incidence with no disinfection in commercial greenhouses (Figure 4), the curved shape of the dynamics of D in both models is closely similar to that of the observed disease incidence (Figure 3A,B and Figure 4).

### 3.3. Estimating Control Effects Using PHLID Model

To simulate the impact of soil disinfection on primary inoculum control, PHLID model C was developed based on model A, with variables *c*_1_ and *c*_2_ adjusted (*c*_1_ = 0.48, *c*_2_ = 1), as shown in Figure 3C. When compared to PHLID models A and C, model A showed a disease incidence rate of 70.0% (as seen in the curve of D, which shows the dynamics of disease plants), while model C showed a disease incidence rate of 56.9% at 127 days after infection and an *R*_0_ value of 2.8. These results suggest that treatment with soil disinfection alone may not be sufficient to effectively control the occurrence of TBC during cultivation (Figure 3A,C).

On the other hand, PHLID model D was developed to simulate the impact of disinfecting contaminated scissors on secondary inoculum control, based on variables *c*_1_ and *c*_2_ adjusted (*c*_1_ = 1, *c*_2_ = 0.12) (Figure 3D). When compared to PHLID models C and D, model D showed a disease incidence rate of 8.4% at 127 days after infection and an *R*_0_ value of 1.1. These results suggest that disinfecting contaminated scissors could significantly inhibit the transmission of *Cmm* and reduce TBC incidence much more effectively than soil disinfection alone (Figure 3C,D).

Furthermore, PHLID model E was developed to simulate the combined effects of soil disinfection and disinfection of contaminated scissors, based on variables *c*_1_ and *c*_2_ adjusted (*c*_1_ = 0.48, *c*_2_ = 0.12) (Figure 3E). Model E showed a disease incidence rate of 4.2% at 127 days after infection and an *R*_0_ value of 0.7, indicating that combining both treatments of soil and scissors disinfections strongly inhibited the transmission of *Cmm* by scissors in greenhouses and demonstrated the best control effects, almost perfectly inhibiting the expansion of TBC (Figure 3E). When compared to the two types of PHLID model E and the observed disease incidence with disinfection in commercial greenhouses (Figure 4), the average observed disease incidence in commercial greenhouses was 21.1% at 127 days, which was higher than the simulation result of model E (Figure 3E and Figure 4).

## 4. Discussion

Previously, the authors developed the HLD model, which is a simulation model of TBC that increases disease based on an SIR model. This model was able to estimate the dynamics of disease incidence caused by infected scissors [3]. In this study, a new PHLID model was developed that estimates the dynamics of disease increase caused by primary infections, including seed-borne and soil-borne cases, as well as secondary infections using infected scissors. TBC is a disease that can be transmitted through both soil-borne and seed-borne routes [2,3,7,8,9]. However, it is not clear which infection route has a stronger effect on the onset of TBC. The results of this study showed that PHLID model A, which considers soil-borne infections and uses the infection rate parameter from Kawaguchi et al. [3], showed slightly more disease incidence than model B, which considers seed-borne infections and uses the infection rate from Hadas et al. [23]. However, both models were similar and would produce almost the same results in commercial greenhouses, suggesting that both infection routes have a similar impact on disease incidence. The results also indicate that pruning using infected scissors as a secondary infection is stronger than primary infection in affecting disease incidence. These findings support the previous reports by Kawaguchi et al. [11] and Kawaguchi and Tanina [9], which suggest that primary inoculum primarily serves as a source of secondary spread. Although controlling the primary inoculum is still important, according to PHLID models C, D, and E, the control effect of soil disinfection alone could not be enough to control TBC occurrence during cultivation, but scissors disinfection and combining both treatments could strongly control it, indicating that preventing the secondary spread is effective and efficient for TBC management. Thus, scissors disinfection should be essentially done in commercial greenhouses with TBC outbreaks.

The PHLID model was developed based on the SEIR model suggested by Madden and van den Bosch [16] and modified for botanical epidemic models. Madden and van den Bosch’s model included parameters for primary inoculum (*P*) and primary infection rate (*v*) [16], with *P* representing the abundance of pathogen inoculum. When *P_0_* units of pathogen inoculum were introduced into a disease-free crop at time (*t* = 0), new infections occurred at a rate of *vSP* during the growing season [16]. This theory was incorporated into our PHLID model and combined with the parameter *c*_1_. However, Madden and van den Bosch’s model was developed as a theory of plant epidemics and was not specifically designed to fit actual and specific plant diseases [16].

This study involved fitting the PHLID model to observe the incidence of TBC in commercial greenhouses. The PHLID model incorporated parameters *c*_1_ and *c*_2_, which represented the control effects of interrupting primary and secondary inoculum transmission and was able to simulate both effects. However, the average observed disease incidence in commercial greenhouses, where both soil disinfection and contaminated scissors treatments were carried out was higher than the simulation result of the PHLID model E. This indicates that the simulation result tended to show a higher expected control effect than the actual effect. The parameters *c_1_* and *c_2_*, were obtained from well-designed experiments, suggesting that the control effects can be estimated accurately [2,3]. Occasionally, growers may not be able to completely disinfect their scissors before each contact with tomato plants during the cultivation period. To prevent any secondary infections caused by contaminated scissors, growers should dip their scissors into a 70% ethanol solution every time after pruning around 50 to 100 tomato plants for cultural practices. However, continuous disinfection may be difficult to maintain due to other practices such as harvest, pesticides, and/or fungicide spraying. Thus, it is important for users of the PHLID model to understand that the control effects estimated by the model may be slightly higher than the actual effects. Nonetheless, the PHLID model remains a useful tool for estimating not only the disease incidence dynamics but also the control effects before planting.

In general, when developing statistical models, a vast amount of data are used to estimate parameters, including meteorological and disease incidence data obtained from governmental and local governmental offices [26,27,28,29]. However, in the case of developing the previous HLD model for TBC, parameters were assumed based on several actual experiments [3]. In this study, the incubation period of TBC was assumed to be 10 days based on experimental results for the population dynamics of *Cmm* in latently infected tomato plants. According to Wang et al. [30], *Cmm* has the ability to migrate both downward and upward in the tomato vascular system, but upward migration via xylem is faster than downward movement. When the stem base was inoculated, *Cmm* was able to migrate further (up to 6 cm from the inoculation site) and reach a higher population of approximately 10^7^ CFU/g plant tissue compared to inoculation at the stem top (which was only 3 cm away from the inoculation site). Our findings that *Cmm* migrated up to 10 cm from the inoculation site at 10 dpi and reached a population of approximately 10^7^ CFU/g plant tissue were consistent with the previous report by Wang et al. [30]. We also found that *Cmm* migrated up to 20 cm from the inoculation site at 10 dpi and reached a population of approximately 10^6^ CFU/g plant tissue in this study. Our previous report [3] indicated that a population of over 1.0 × 10^6^ CFU/g of *Cmm* in plant tissue is sufficient to infect plants and cause TBC symptoms. Based on these findings, we assumed an incubation period of 10 days for TBC. Our findings suggest that *Cmm* is capable of spreading widely within the plant, which highlights the importance of early detection and effective control measures for preventing the spread of TBC. As mentioned previously, it is necessary to disinfect infected scissors (and perform soil disinfection if possible) in order to prevent the spread of infection.

## 5. Conclusions

This is the first study to develop a PHLID model of TBC using biological parameters obtained from precise inoculation experiments, including the incubation period of TBC. Utilizing an epidemiological model such as PHLID can not only enhance our understanding of the relationship between increasing numbers of diseased plants, but also simulate the effectiveness of control measures, either alone or in combination, during cultivation. This could be beneficial for growers in making informed decisions on controlling TBC based on their cultivation timeline.

## Figures and Tables

**Table 1:** Parameters used in the PHLID model of tomato bacterial canker transmission.

Parameter	Description	Variable	Reference
*B*	Infection rate per contact (inoculated 10^6^ cells/mL of *Cmm*)	0.706	[3]
*k*	Number of contacts per day	0.286	[3]
*d*	Duration of infection (days)	12	[3]
1/*e*	Incubation period (days)	10	Figure 2
*v_soil_*	Infection rate by infected seeds as primary inoculum	0.067	[9]
*v_seed_*	Infection rate by infected seeds as primary inoculum	0.04	[23]
*c*_1_	Integrated relative risk of soil disinfection from five independent field experiments	0.48	[24]
*c*_2_	Integrated relative risk of scissors disinfection from four independent field experiments	0.12	[3]

## Data Availability

The data presented in this study are available on request from the corresponding author.
